# Ultrasonography, an operator-dependent modality versus dual-energy computed tomography (DECT) in the detection of chondrocalcinosis: with regard to Tanikawa et al.’s study

**DOI:** 10.1186/s13018-018-0953-4

**Published:** 2018-10-16

**Authors:** Angel Checa

**Affiliations:** Lourdes Medical Center Burlington County, Willingboro, NJ 08046 USA

## To the editor

I read with interest the article by Tanikawa et al. [1] that appeared recently in the Journal of Orthopedics Surgery Research. Greatly, this is an interesting paper about the role of dual-energy computed tomography (DECT) in the detection of chondrocalcinosis. However, I believe authors gave much more emphasis to the operator-dependent, a well-known obstacle in musculoskeletal sonography.

Certainly, musculoskeletal ultrasonography as many other diagnostic modalities requires several months of training. Good multiplanar sonographic images rely on adequate understanding about the machine tributes, optimization of the image, and a precise transducer positioning. Skilled operators will be able in many instances to avoid or correct common artifacts and pitfalls. Also, profound knowledge about anatomy, anatomical variants, biomechanics, and elementary lesions are crucial in the interpretation of a musculoskeletal sonogram.

Tanikawa et al. [1], however, did not mention in their article about the availability of ultrasound and the possibility to scan several joints with lower expenditures at the same visit. On the other hand, while this paper has appeared, mounts of publications demonstrate that ultrasound is an excellent modality in the diagnosis of chondrocalcinosis. Ultrasonography allows us to get detailed additional information other than the presence of calcium deposits. It can reveal changes on the hyaline cartilage, fibrocartilage, synovium, tendons, and bone [2].

Deposits of calcium as small as 0.8 mm have been detected in patients with no radiographic chondrocalcinosis [2]. A crystal-minimal distance to the cartilage surface as a predictor of crystal shedding and the distinction of two different sonomorphologic patterns of chondrocalcinosis (with and without radiographic evidence) seems attractive avenues for future investigations [2].

Concerning about the operator-dependent aspect in ultrasonography and contemplating the data is limited in this regard, several reports using ultrasound have showed that the learning curve for some particular musculoskeletal lesions is relatively short [3–5]. Gutierrez et al. [4] found that rheumatologists with limited experience in ultrasound, after a week of training, were able to detect and interpret sonographic change characteristic of monosodium urate crystal deposits in patients with gout. Therefore, some authors stated that the criticism against musculoskeletal ultrasound as an operator-dependent technique should be attenuated [5].

In summary, after considering the cost of ultrasound, the possibility to be done bedside or in the office and the ability to perform as many joints as needed, two questions emerge. First, is the DECT available for a daily practice at the office? Second, is DECT rather than ultrasonography a nonoperator-dependent tool?

## References

[1] Tanikawa H, Ogawa R, Okuma K, Harato K, Niki KS, Nagura T (2018). Detection of calcium pyrophosphate crystal in knee meniscus by dual-energy computed tomography. J Orthop Surg Res.

[2] Checa A, Hussain H, Russell E (2013). Quantitative echogenicity of small intra-articular deposits of calcium, hyaline cartilage thickness and sonographic aspects of the synovium in patients without radiographic chondrocalcinosis. Osteoarthr Cartil.

[3] D’Agostino MA, Maillerfert JF, Said-Nahal R, Breban M, Ravaud P, Dougados M (2004). Detection of small joint synovitis by ultrasonography: the learning curve of rheumatologists. Ann Rheum Dis.

[4] Gutierrez M, Di Geso L, Rovisco J, Di Carlo M, Ariani A, Fillipucci E, Grassi W (2013). Ultrasound learning curve in gout: a disease-oriented training program. Arthritis Care Res.

[5] Ohrndorf S, Naumann L, Grundey J, Scheel T, Scheel AK, Werner C, Backhaus M (2010). Is musculoskeletal ultrasonography an operator-dependent method or a fast reliably teachable diagnostic tool? Interreader agreements of three ultrasonographers with different training levels. Int J Rheumatol.

[6] Ward IM, Scott JN, Mansfield LT, Battafarano DF (2015). Dual-energy computed tomography demonstrating destructive calcium pyrophosphate deposition disease of the distal radioulnar joint mimicking tophaceous gout. J Clin Rheumatol..

[7] Kim HR, Lee JH, Oh J, Kim NR, Lee SH (2012). Clinical images: detection of gouty arthritis in the atlantoaxial joint using dual-energy computed tomography. Arthritis Rheum..

[8] Yoo Y, Seo YJ, Huh M, Yoo JH, Yun KH, Kim SJ (2011). Gout and coexisting pseudogout in the knee joint. Knee Surg Sports Traumatol Arthrosc..

[9] Kim HR, Lee JH, Kim NR, Lee SH (2014). Detection of calcium pyrophosphate dihydrate crystal deposition disease by dual-energy computed tomography. Korean J Intern Med..

