# Supplementary material for: Ultrasonography, an operator-dependent modality versus dual-energy computed tomography (DECT) in the detection of chondrocalcinosis: with regard to Tanikawa et al.’s study
Source: J Orthop Surg Res. 2018 Oct 16;13:255. doi: 10.1186/s13018-018-0953-4 (PMC6192358; doi:10.1186/s13018-018-0953-4)
Supplement: Related texts

# Reply: Answer to the letter

**Authors:** Hidenori Tanikawa

**Affiliations:** Saiseikai Yokohamashi Tobu Hospital, Yokohama, Kanagawa Japan

The answer to the first question is that DECT is not suit for a daily practice at the office. I agree that the ultrasonography is able to scan several joints at the same visit as well as to scan blood flow at the same time. DECT is more expensive, more invasive, and less available compared to ultrasonography. However, DECT offers several advantages over ultrasonography. First, the DECT can distinguish Calcium pyrophosphate dihydrate (CPPD) crystals from sodium urate crystals, which information is helpful for diagnosis. Ward IM reported a case using DECT to diagnose tumoral CPPD mimicking tophaceous gout [6]. DECT may be useful for a case of gout and coexisting pseudogout in the same joint [7]. Second, DECT can detect crystals at deep complex structures. Kim HR documented that DECT was able to detect urate deposits in the atlantoaxial joint, as well as to detect CPPD crystals in the anterior cruciate ligament or posterior cruciate ligament [8, 9]. Finally, we can quantify the pathological lesion three-dimensionally using DECT. This information may be useful in monitoring therapeutic response. The answer to the second question is that DECT is not operator-dependent. The settings of parameters for postprocessing may be different in each hospital, and generated images sometimes contain artifacts especially detecting CPPD crystals, but basically, DECT is not operator-dependent.
